# Primary Diffuse Large B‐Cell Lymphoma of the Central Nervous System—Outcomes in Finland: A Nationwide Population‐Based Study

**DOI:** 10.1002/jha2.70021

**Published:** 2025-05-28

**Authors:** Mikko Moisala, Ilja Kalashnikov, Niklas Haataja, Sirpa Leppä, Marjukka Pollari

**Affiliations:** ^1^ Department of Neurology Tampere University Hospital Tampere Finland; ^2^ Research Programs Unit and University of Helsinki Helsinki Finland; ^3^ Department of Oncology University of Helsinki Helsinki Finland; ^4^ Department of Oncology Helsinki University Hospital Comprehensive Cancer Centre Helsinki Finland; ^5^ Department of Oncology Tays Cancer Center Tampere University Hospital Tampere Finland

## Abstract

Primary central nervous system lymphoma (PCNSL) is a rare and aggressive malignancy commonly presenting with a rapid disease course and poor prognosis. Recent clinical trials have indicated improved treatment outcomes in a highly selected patient population. However, real‐world data focusing on long‐term, population‐based outcomes remain largely unexplored. We analyzed trends in relative survival (RS) in patients diagnosed with PCNSL in Finland from 1995 to 2018. We identified 718 PCNSL patients from the comprehensive Finnish Cancer Registry (FCR) (51% males, median age 67.8 years). For the entire cohort, 5‐year overall survival (OS) and RS rates were 21% and 22%, respectively. The 2‐year RS was 39% for patients younger than 75 years and 14% for older patients. A gradual increase in the 2‐year RS rate was observed over successive chronological diagnostic periods. Age above 75 years at diagnosis (HR 3.65, 95% CI: 2.73–4.89) and diagnosis during a calendar period of 1995–2006 (HR 1.30, 95% CI: 1.10–1.53) were associated with a significantly increased risk of death. An increase in the number of patients diagnosed with PCNSL during the study period was confirmed, and the prognosis of patients diagnosed after the age of 75 years continues to be dismal.

## 1 Introduction

Primary diffuse large B‐cell (DLBCL) lymphoma of the central nervous system (CNS) (PCNSL) is a rare immune‐privileged malignancy, affecting the brain, spinal cord, leptomeninges, and/or eyes in the absence of systemic manifestation. PCNSL differs from other lymphoma entities including systemic DLBCL, as it is isolated to the structures of the CNS only. PCNSL accounts for 2.4%–3.0% of all brain tumors and less than 1% of all lymphomas [1, 2]. Over 90% of PCNS lymphomas are histologically classified as DLBCLs; other histologies like Hodgkin lymphoma or low‐grade B‐cell lymphomas including marginal zone lymphoma and mantle cell lymphoma are rarely seen [3]. The incidence of PCNSL is 0.47 per 100,000 population, increasing among individuals older than 60 years [4, 5].

The clinical course of PCNSL is highly aggressive, and the outcome is poor. Recent population‐based studies have shown a positive trend in survival with modern treatments including induction with high‐dose methotrexate‐based (immuno‐)chemotherapy and consolidation with high‐dose chemotherapy followed by autologous stem‐cell transplantation (HDCT/ASCT) [6, 7]. However, this positive trend does not involve the elderly patient population, and the survival of PCNSL patients older than 70 years of age has not improved during the last four decades [8]. Challenges in identifying the optimal treatment options for elderly patients are due to a limited amount of Phase 3 studies and the lack of elderly patients in prospective studies, despite the characteristically high median age at diagnosis.

We conducted a nationwide population‐based study in patients diagnosed with PCNSL between 1995 and 2018 in Finland, to uncover possible changes in the age of disease onset and excess mortality. We included only patients with histologically confirmed DLBCL.

## 2 Methods

### 2.1 Data Sources

The Finnish Cancer Registry (FCR) is a national institute focused on statistical and epidemiological research, tasked with recording data on all new cancer diagnoses and treatments. Cancer registration to FCR has been mandatory for Finnish physicians, hospitals, and pathology laboratories since 1961. Each case is given a unique personal identity code, enabling linkage with national registries and reliable patient follow‐up. Since 2007, cancer subtype coding has adhered to the International Classification of Diseases for Oncology, 3rd Edition (ICD‐O‐3), in line with the World Health Organization's Classification of Tumors of Haematopoietic and Lymphoid Tissues, 4th edition [9, 10]. From 1953 to 2006, cancer morphology was coded using a modified version of the Manual of Tumor Nomenclature and Coding, with codes converted to ICD‐O‐3 in 2007 [11, 12]. The FCR continuously updates information on the vital status and residence of cancer patients from the Population Information System managed by the Digital and Population Data Services Agency. The registry boasts high coverage, with a recent study estimating 94% completeness for non‐Hodgkin lymphomas and 99% morphological verification of cases [13, 14].

### 2.2 Patients

Patients with PCNSL were extracted from the FCR using ICD‐O‐3 codes C71.0–C71.9 for brain tumor location and 9680/3 and 9684 for DLBCL or 9590/3, 9591/3 for lymphoma not otherwise specified (NOS) histology. In circumstances where tumor histology or the lack of systemic involvement could not be verified from FCR data, clinical reports were reviewed to update and clarify the FCR database information. In unclear cases, additional information was requested from the pathology laboratories. DLBCL histology and PCNSL diagnosis were confirmed from the free‐text part of the pathology reports. Other, more uncommon PCNSL lymphoma, including marginal‐zone lymphoma, were not included to gain uniform and representative data.

Patient selection was restricted to those diagnosed between 1995 and 2018. We collected demographic, diagnostic, and follow‐up data on individual patients until December 31, 2022, excluding patients (*n* = 14) diagnosed with concurrent systemic lymphoma, histology other than DLBCL, CNS DLBCL diagnosis only from autopsy/death certificate, or OS less than 1 day. Individual patient data were gathered for all PCNSL patients, including sex, date of birth, time of PCNSL diagnosis, follow‐up time, vital status at the end of follow‐up, and time of death if the patient had died during the follow‐up period. The study cohort was followed from January 1, 1995 until December 31, 2022. No patient was lost to follow‐up before the end of the study period. The general population mortality rates of Finland were obtained from the Human Mortality Database.

The study was approved by the National Institute for Health and Welfare (Dnro THL/1441/5.05.00/2019), Statistics Finland (Dnro TK‐53‐1172‐19), and Helsinki University Hospital Institutional Review Board (HUS/259/2021‐56).

### 2.3 Definitions and Statistical Analyses

The start of follow‐up was defined as the date of PCNSL diagnosis. The Kaplan–Meier method was utilized to estimate overall survival (OS). Hazard ratios (HRs) for overall mortality were estimated using multivariable Cox regression models. Relative survival (RS) was estimated using the Ederer II method with internal age standardization (age groups: 0−44, 45−54, 55−64, 65−74, and ≥75 years) and monthly intervals for collapsing the data [15]. Follow‐up time was split into monthly intervals. Age‐specific RS was estimated for three age groups by age at diagnosis (0−54, 55−74, and ≥75 years). A complete analysis was based on all person‐time and deaths in 1995−2022. Period analyses were carried out for 1995−2006 and 2007−2018, with left‐truncated data for the later time period [16]. To gain a more nuanced statistical analysis of recent years and the impact of modern treatments, the more recent time period was divided into 3‐year subcohorts: 2007–2009, 2010–2012, 2013–2015, and 2016–2018, for additional analyses. Age at diagnosis was modeled as a linear continuous variable, and year of diagnosis as a linear continuous or categorical variable.

To estimate HRs for excess mortality, we used multivariable flexible parametric survival models [17]. Age at diagnosis, calendar period of diagnosis, and sex were included in the multivariate model concerning total mortality. Expected mortality was based on the general population mortality rates in Finland by age group with 1‐year intervals, calendar year, and sex. The baseline excess HR was modeled with 4 degrees of freedom [18]. The Wilcoxon rank‐sum test was used to compare age at diagnosis between groups.

All statistical analyses were conducted using R, version 2024.04.2 (R Foundation for Statistical Computing, Vienna, Austria), with the packages survival 3.7‐0, relsurv 2.2‐9, flexsurv 2.3, rstpm2 1.6.3, dplyr 1.1.4, ggplot2 3.5.1, and Epi 2.47.1.

## 3 Results

### 3.1 Patients

We identified 732 patients diagnosed with PCNSL between January 1995 and December 2018. Characteristics of the 718 patients included in the analysis are shown in Table 1. The median age at diagnosis was 67.8 years (interquartile range [IQR]: 60.7–74.9 years). Females were slightly older than males, with a median age of 68.9 versus 66.2 years (*p* = 0.002). The median follow‐up time was 6.4 months (IQR: 2.1–40.8 months) for the whole cohort and 7.6 years (IQR: 5.3–11.5 years, range: 4.1–25.3 years) for the patients alive at the end of the follow‐up period.

**TABLE 1:** Characteristics of patients, and overall survival of different age groups and time periods of diagnosis.

	*n* (%)	Median follow‐up time (days)	1‐year RS (95% CI)	2‐year RS (95% CI)	5‐year RS (95% CI)	2‐year OS (95% CI)
**Total**	718	194	38% (35–42)	32% (29–36)	22% (19–25)	31% (28–35)
**Sex**						
Male	367 (51.1)	212	38% (33–43)	32% (27–37)	22% (18–27)	31% (27–36)
Female	351 (48.9)	188	39% (34–45)	33% (28–38)	22% (18–27)	32% (27–37)
**Age groups**						
<55	98 (13.6)	798	57% (48–68)	50% (41–61)	39% (31–50)	50% (41–61)
55–74	452 (63.0)	233	42% (38–47)	35% (31–40)	23% (20–28)	35% (31–39)
≥75	168 (23.4)	76	17% (12–23)	13% (9–19)	7% (4–13)	12% (8–18)
**Calendar period**						
1995–2006	253 (35.2)	160	34% (29–40)	30% (25–36)	15% (11–20)	29% (24–35)
2007–2018	465 (64.8)	219	41% (37–46)	33% (29–38)	26% (22–30)	33% (29–37)
2007–2009	99 (13.8)	125	35% (26–45)	30% (22–40)	23% (16–33)	29% (22–40)
2010–2012	106 (14.8)	130	39% (31–50)	30% (22–40)	22% (15–31)	29% (22–39)
2013–2015	110 (15.3)	125	38% (30–48)	30% (22–40)	23% (16–33)	29% (22–39)
2016–2018	150 (20.9)	157	49% (41–57)	41% (34–50)	32% (25–41)	40% (33–49)

Abbreviations: CI, confidence interval; OS, overall survival; RS, relative survival.

**FIGURE 1:**
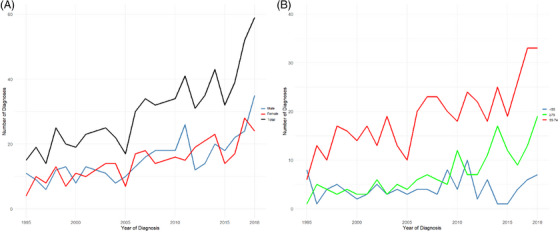
Number of diagnoses per year in the entire cohort and in different genders (A), and according to age at diagnosis (B). The age groups are divided into three categories: <55, 55–74, and ≥75 years.

The change in the number of new PCNSL diagnoses per year is visualized according to sex and age group in Figure 1A,B. A significant increase in the amount of yearly PCNSL diagnoses was confirmed, but only among patients older than 54 years. The average change in the number of new diagnoses was 9.3% (95% CI: 1.00–1.84, *p* < 0.001) annually for the whole cohort. There was no statistical difference in the number of new diagnoses per year between males and females.

### 3.2 Survival

**FIGURE 2:**
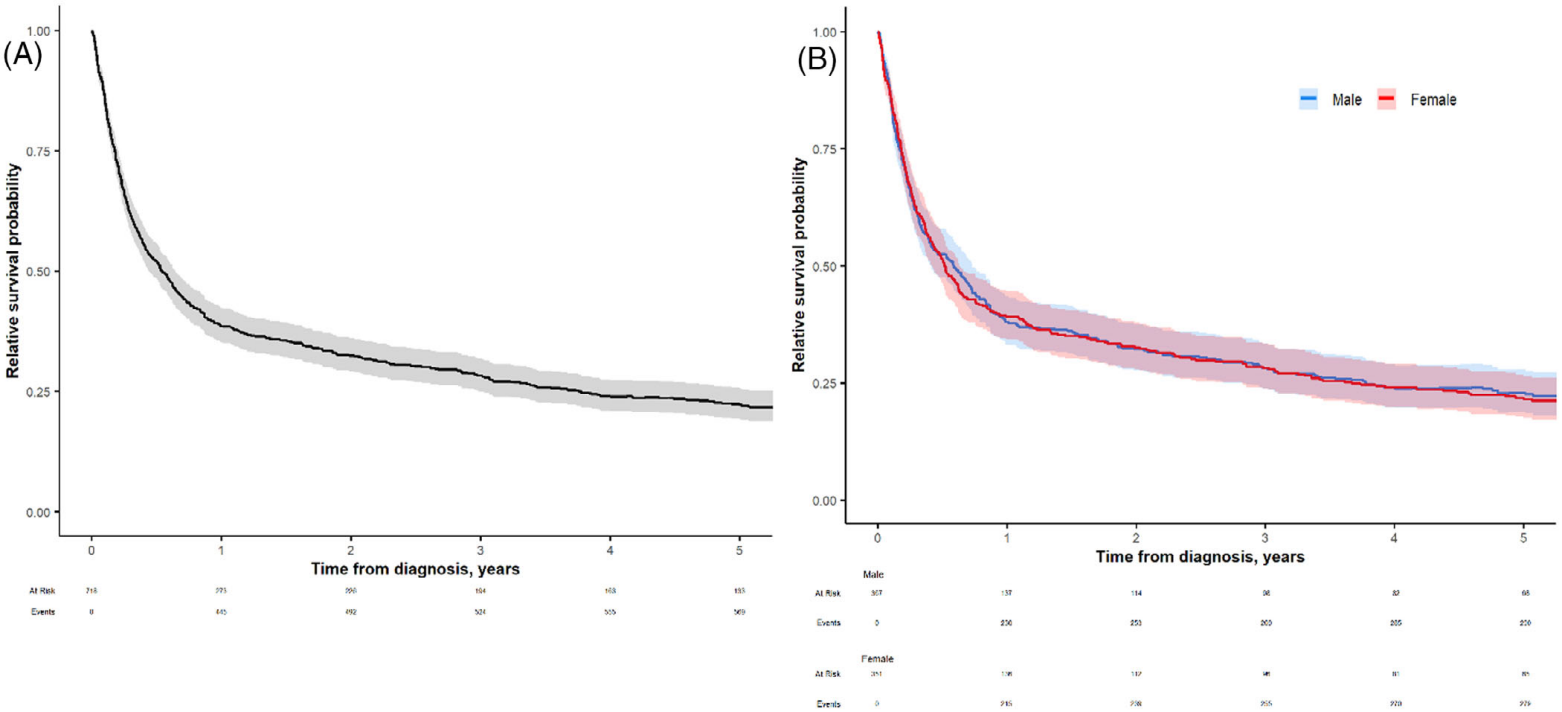
Five‐year relative survival probability for the whole cohort (A) and in different genders (B).

Among the 718 reported cases, 626 deaths were recorded. The total follow‐up time was 1813 person‐years, and the crude mortality rate was 345.2/1000. For all patients, the 1/2/5‐year OS was 38% (95% CI: 35%–42%)/31% (95% CI: 28%–35%)/21% (95% CI: 18%–24%), respectively. The 5‐year RS for all patients was 22% (95% CI: 19%–25%). There was no significant difference in OS or RS between males and females. RS for all patients is shown in Figure 2A (OS in Figure S1A) and stratified by gender in Figure 2B (OS in Figure S1B).

**FIGURE 3:**
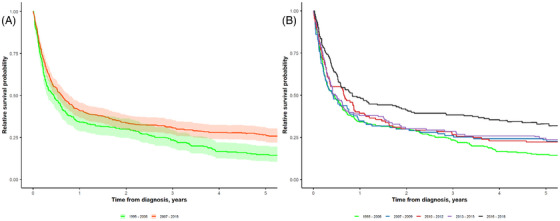
Relative survival probability according to two different diagnostic time periods (A), and further stratified according to five different diagnostic time periods (B).

A group analysis was performed by dividing the patients into five groups according to year of diagnosis: 1995–2006, 2007–2009, 2010–2012, 2013–2015, and 2016–2018. The number of patients in each group, and 1/2/5‐year RS and 2‐year OS rates are shown in Table 1, and Kaplan–Meier estimates for RS are shown in Figure 3B (OS in Figure S2A). A significant increase was observed in the 2‐year RS in the last calendar period of 2016–2018 reaching 41%, while other time periods between 1995 and 2015 had a common 2‐year RS of 30%. The gradual improvement in the latest calendar periods was mainly observed among patients younger than 75 years at diagnosis.

Most recent time period of 2016–2018 was associated with a lower risk of mortality (HR 0.51, 95% CI: 0.40–0.65, *p* ≤ 0.001) compared with 1995–2007 (Table S1). With broader groups for diagnostic time periods, 2007–2018 was associated with a lower risk of death (HR 0.77, 95% CI: 0.66–0.91, *p* = 0.002) compared to 1995–2006 (Figure 3A, Table 2).

**TABLE 2:** Hazard ratios for excess mortality.

	Unadjusted			Adjusted		
Variable	HR	95% CI	*p*‐value	HR	95% CI	*p*‐value
**Sex**						
Male	1.00 (ref)			1.00 (ref)		
Female	0.99	0.85–1.16	0.9	0.90	0.77–1.11	0.21
**Age group**						
0–54	1.00 (ref)			1.00 (ref)		
55–74	1.79	1.38–2.32	<0.001	1.91	1.47–2.48	<0.001
≥75	3.65	2.73–4.89	<0.001	4.36	3.23–5.88	<0.001
**Year of diagnosis**						
1995–2006	1.00 (ref)			1.00 (ref)		
2007–2018	0.77	0.66–0.91	0.002	0.67	0.57–0.79	<0.001

Abbreviations: CI, confidence interval; HR, hazard ratio.

**FIGURE 4:**
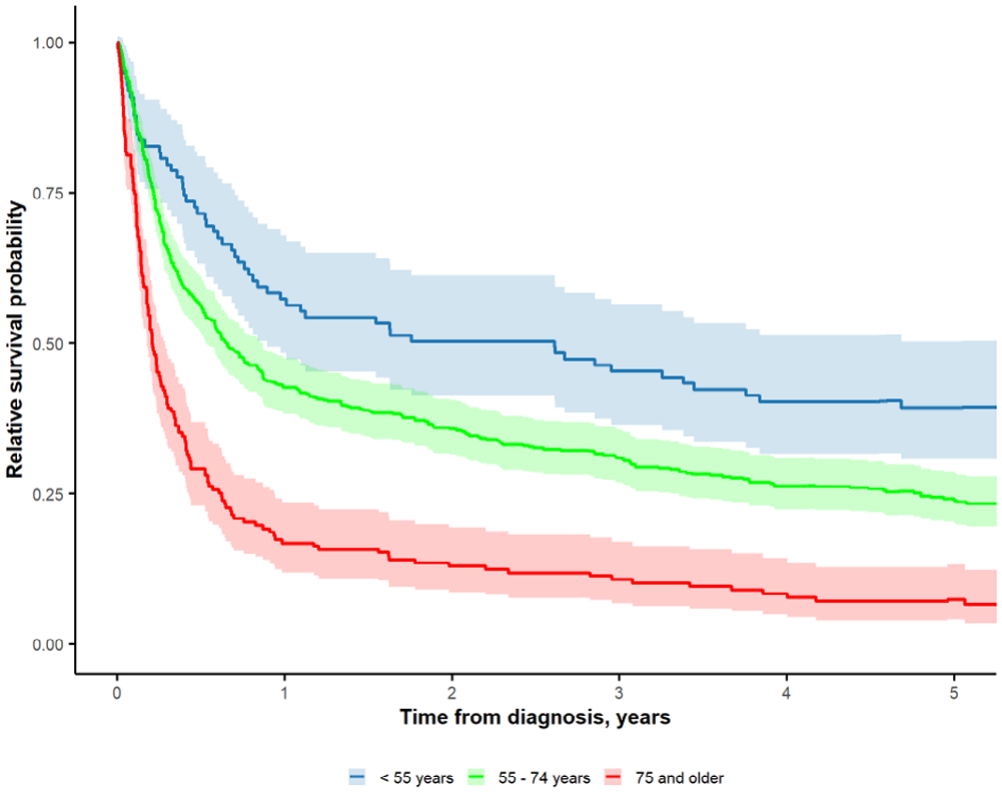
Survival probability in different diagnostic age groups of <55, 55–74, and ≥75 years.

Another group analysis was performed by dividing the patients into three groups by age at diagnosis: 0–54, 55–74, and 75 years and older. The number of patients in each group and 1/2/5‐year RS and 2‐year OS rates are shown in Table 1. Kaplan–Meier curves for RS are shown in Figure 4A, OS in Figure S3A. Comparing 2‐year RS between age groups, patients diagnosed before the age of 75 years (*n* = 548, 77%) demonstrated an RS of 29% (95% CI: 26%–32%), while those diagnosed at 75 years and older exhibited a significantly lower RS of 14% (95% CI: 10%–21%). Patients aged 55–74 years at diagnosis had an unadjusted HR of 1.79 (95% CI: 1.38–2.32, *p* ≤ 0.001), while those aged 75 years and older had an HR of 3.65 (95% CI: 2.73–4.89, *p* ≤ 0.001). Adjusted analyses showed HRs of 1.91 (95% CI: 1.47–2.48, *p* < 0.001) for the 55–74 age group and 4.36 (95% CI: 3.23–5.88, *p* < 0.001) for those 75 and older.

### 3.3 Excess Risk of Death

Adjusted and non‐adjusted HRs for genders, different age groups, and time periods of diagnosis are shown in Table 2.

In non‐adjusted analyses, sex was not associated with excess mortality. A diagnostic age of 75 years and older correlated with increased mortality risk, 3.65 (95% CI: 2.73–4.89). The last calendar period of diagnosis, 2016–2018, was associated with a reduced mortality risk, HR 0.63 (95% CI: 0.50–0.79) compared to 1995–2006.

Adjusted for age and diagnosis year, sex was not associated with excess mortality. When adjusted for diagnosis year, older age groups were associated with a significant increase in mortality risk. After age adjustment, a significant reduction in mortality risk was observed in patients diagnosed between 2010 and 2018, compared to 1995–2006, decreasing to 0.52 (95% CI: 0.41–0.65) in diagnoses made between 2016 and 2018.

## 4 Discussion

In this nationwide population‐based study, we found that across the last decades, more PCNSLs were diagnosed annually, specifically in the older population. A clear increase in RS was confirmed in the most recent time period. Regarding the different risk factors, sex was not a standalone risk, but older age and an earlier calendar period of diagnosis were found to be associated with reduced OS and RS rates. Even after age adjustment, the most recent time period of diagnosis was associated with a lower mortality risk. These findings align with other population‐based studies in Europe and the United States [4, 8, 19, 20]. A previous report on patient outcomes in Finland showed poor survival in patients diagnosed in 2007–2017; here, we expand the timeline to highlight the gradual improvement in survival in recent years [21].

Median age of 67.8 (IQR: 60.7–74.9) years at diagnosis in this cohort was consistent with other recent population‐based studies with immunocompetent patients [4, 20, 22–24] but slightly older than in Italy [19]. Even though the median age at diagnosis did not differ between the compared diagnostic periods, year‐by‐year comparison presents clues of a rising median age at diagnosis (median age at diagnosis 54.2 years in 1995 [*n* = 15] and 70.7 years in 2018 [*n* = 59]). Mortality was high throughout the study period, but a trend toward a better RS appeared in the latest diagnostic periods. Looking at the age groups specifically, we found that the number of patients older than 75 years at diagnosis increased in the latest calendar periods, but despite this change in demographics, the RS substantially improved.

Available and commonly used treatment modalities have gone through an overhaul during the study period. At the start of the study period, radiotherapy in combination with corticosteroids was common practice [25, 26]. Around the year 2000, high‐dose methotrexate (HD‐Mtx)‐based chemotherapy and intravenously and/or intrathecally administered injections became increasingly more common, in addition to radiotherapy [27, 28, 29]. In 2015, the Nordic PCNSL regimens consisting of HD‐Mtx, procarbazine, vincristine, and high‐dose cytarabine (HD‐AraC) were developed by NLG as a modified version of the “Bonn protocol,” and also included a treatment regimen tailored for 66–75‐year‐old patients [27, 30]. No treatment regimens have been universally accepted for the treatment of over 75‐year‐old patients. The concept of induction and consolidation treatment in PCNSL was conceived and became common practice during the study period [31, 32]. In 2016, the MATRix regimen consisting of HD‐Mtx, cytarabine, thiotepa, and rituximab was found to be effective and safe for healthy, young patients as an induction treatment [33]. HD‐Mtx has been shown to have severe adverse effects on all age groups, especially in elderly patients, but robust and specific risk factors for severe Mtx‐related toxicity have not been found [34, 35]. HDCT/ASCT initially emerged as an alternative treatment to whole‐brain radiotherapy for recurrent or refractory disease. Later, it became the preferred first‐line consolidation treatment [36, 37, 38]. Treatment guidelines are similar nationwide in Finland. HD‐Mtx‐based therapy has been used throughout the study period, MATRix treatments since 2015, and ASCT consolidation treatments beginning in Finland circa 2016.

We found that through the study period (1995–2018), the number of new PCNSL diagnoses annually increased, specifically among the older population. Our finding is consistent with recent studies conducted in Europe and the United States. Even though more PCNSLs are diagnosed in elderly patients, the mortality risk has reduced in the later diagnostic calendar periods. This change in PCNSL patient demographics has happened simultaneously with the increasing amount of overall brain tumor diagnoses in the older population and it has been speculated to coincide with changes in the clinical setting leading to a higher amount of confirmed PCNSL cases, and more thorough clinical workup in the elderly population [4, 39, 40]. Computed tomography and the use of magnetic resonance imaging have become better available and utilized for neurological symptoms. Protocols for intracranial tumor biopsies and general anesthesia have been developed to include elderly patients with good functional ability reserves. As the average life expectancy in Finland has changed from 76.4 years in 1995 to 81.6 years in 2018, and comorbidities are treated more effectively, we have a healthier elderly population with fewer contraindications to efficacious lymphoma treatments [4, 8, 19, 20, 41, 42]. There are also reports suggesting that increased usage of immunosuppressive medications to manage autoimmune disorders in the elderly population might be driving the incidence higher [5].

The trend of increasing PCNSL diagnoses can also be speculated to be due to improved usage and reporting of cancers to the Finnish Cancer Registry. However, the registry has been recently estimated to include 94% of all non‐Hodgkin lymphomas diagnosed in Finland during the years 2009–2013, and registration of new cancer diagnoses has been mandatory and common practice for pathology and oncology clinics throughout Finland since 1961, so we suspect the possibility of underreporting the lymphoma cases to FCR in the earlier part of the study period to be negligible [14]. The compulsory reporting of cancer diagnoses to the FCR provides a trustworthy tool for population‐based studies, as the cohort consists of a truly consecutive population. Although the data are not exhaustive and do not provide linkage to other registries and other health data, it offers comprehensive and reliable information on accurate diagnoses, time of diagnosis, and mortality data at a national level.

The role of immunosuppression is multifaceted in its relationship with lymphoma. There are reports of an increase in lymphoma incidence in the elderly population. A simultaneous increase is recognized in the usage of immunosuppressive treatments for autoimmune diseases in all age groups. Clinical workup leading to the diagnosis of autoimmune diseases or malignant tumors has become more widely available and efficacious during the study period, with better availability for older patients. More immunosuppressive agents are used for autoimmune diseases or post‐organ transplantation. These changes could cause an additive risk in lymphoma incidence. The role of HIV/AIDS in lymphoma etiology is small in Finland, and because the FCR does not collect data on HIV status, that information was not available for this study [5, 20, 43–45].

We estimated OS and RS probabilities in different subgroups. The improved RS in the later diagnostic time periods suggests a better prognosis due to modern treatments, considering the overall health and life expectancy differences between the different calendar periods. With a steep ascent in the amount of PCNSL cases specifically in the elderly population, the elevation of RS estimates and the reduction in HRs in the later diagnostic time periods suggest refined treatment selection and usage for elderly PCNSL patients. However, the outcome of elderly patients is consistently poor, with a 5‐year RS of 9% in patients older than 75 years. Sex is not a standalone risk factor for mortality in PCNSL, and the non‐significant trend suggested by the adjusted analyses (HR 0.90, *p* = 0.21) is believed to be affected by the slightly older median age of female patients and statistical chance, rather than actual biological differences in lymphoma survival.

In this nationwide cohort, early mortality is high throughout the study period, but a statistically significant improvement in survival was found with the latest diagnostic time period 2016–2018. A smaller positive trend of long‐term survival was seen also in other diagnostic calendar periods after 2007, compared with 1995–2018. This may reflect the effect of modern induction and consolidation treatments. A small proportion of patients (*n* = 92, 12.8%) survived until the end of the follow‐up period, with a median follow‐up time of 7.6 years, in striking contrast with 6.4 months for the whole cohort.

In conclusion, we found an increase in the number of yearly incidents of PCNSL diagnoses in the elderly population. While the prognosis for PCNSL remains poor, survival in younger patients is significantly better compared to elderly age groups. Later calendar periods of diagnosis were associated with better survival. The prognosis for elderly patients remains dismal.

## Author Contributions

Marjukka Pollari, Sirpa Leppä, and Ilja Kalashnikov designed the study. Mikko Moisala, Niklas Haataja, and Ilja Kalashnikov collected the data. Mikko Moisala and Ilja Kalashnikov analyzed the data. Mikko Moisala wrote the manuscript. All authors reviewed and approved the final version of the manuscript.

## Ethics Statement

The study was approved by the National Institute for Health and Welfare (Dnro THL/1441/5.05.00/2019), Statistics Finland (Dnro TK‐53‐1172‐19), and Helsinki University Hospital Institutional Review Board (HUS/259/2021‐56).

## Consent

The study is a registry study and patient consent was not obtained.

## Conflicts of Interest

Sirpa Leppä declares the following competing financial interests: consultancy fees from Abbvie, BMS, Genmab, Gilead, Incyte, Roche, and Sobi, all outside the submitted work; honoraria from Abbvie, Gilead, Incyte, and Sobi; research grants from Bayer, Celgene/BMS, Hutchmed, Genmab, Novartis, and Roche, all outside the submitted work. Marjukka Pollari declares the following competing financial interests: consultancy fees from Abbvie, Gilead, Roche, Serb, and Sobi, all outside the submitted work; honoraria from Abbvie, Incyte, Lilly, and Sobi.

## Clinical Trial Registration

The study is a registry study and not registered as a clinical trial.

## Supporting information

Supporting Information

Supporting Information

Supporting Information

Supporting Information

## Data Availability

The data that support the findings of this study are available from the corresponding author upon reasonable request.
