# Supplementary figures and images for: Primary Diffuse Large B‐Cell Lymphoma of the Central Nervous System—Outcomes in Finland: A Nationwide Population‐Based Study
Source: EJHaem. 2025 May 28;6(3):e70021. doi: 10.1002/jha2.70021 (PMC12118592; doi:10.1002/jha2.70021)

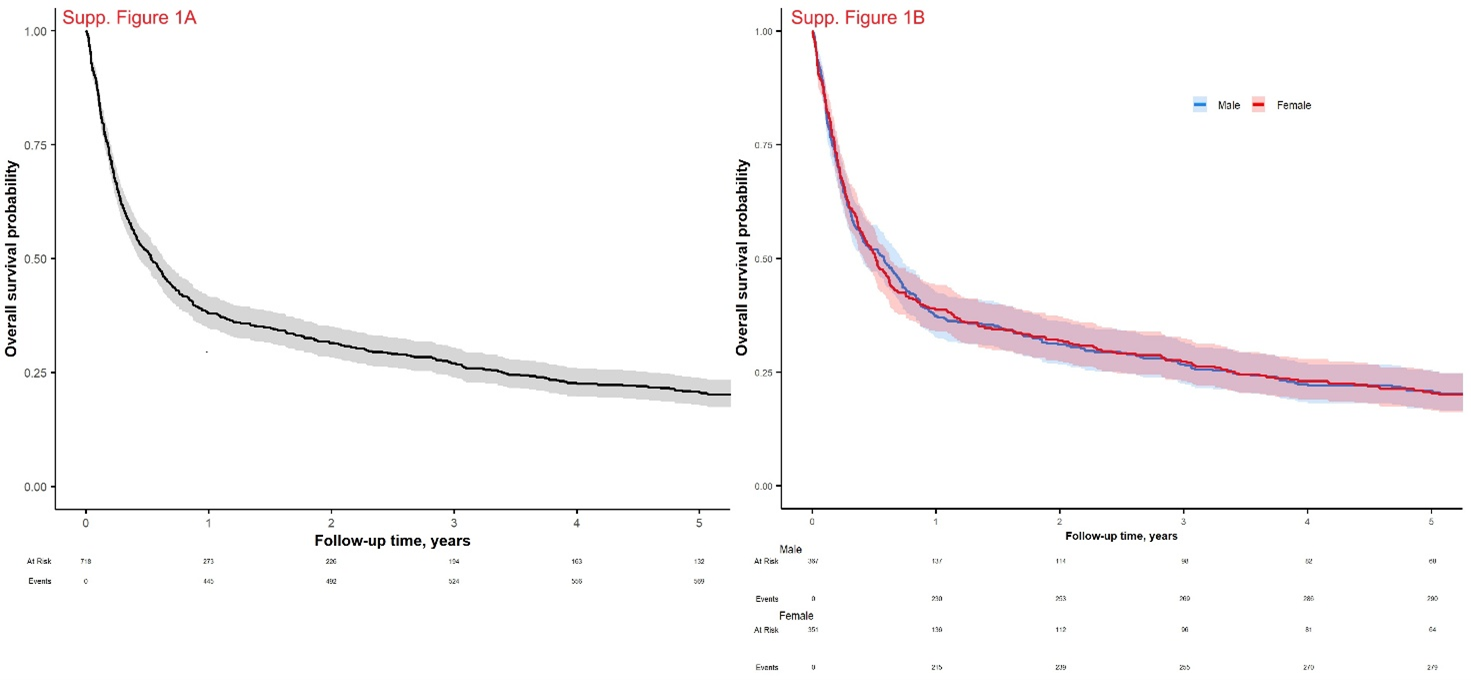

Supplement: Supplementary file 1 — Supporting Information [file JHA2-6-e70021-s004.png]

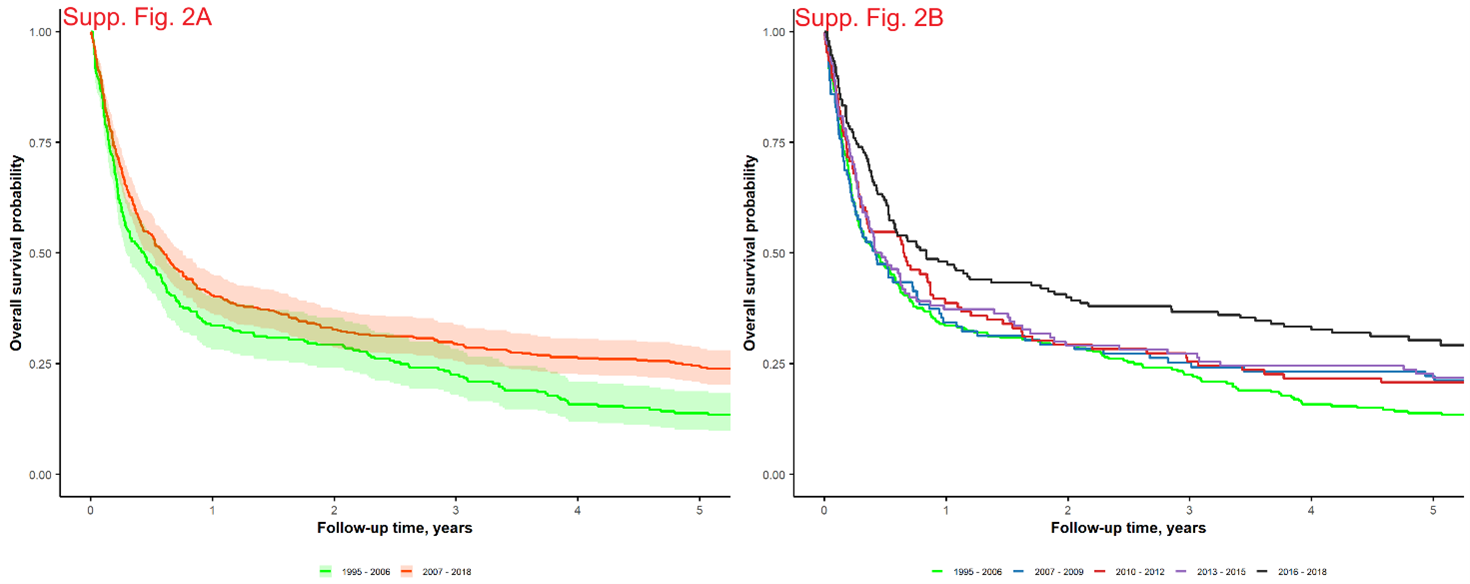

Supplement: Supplementary file 2 — Supporting Information [file JHA2-6-e70021-s001.png]

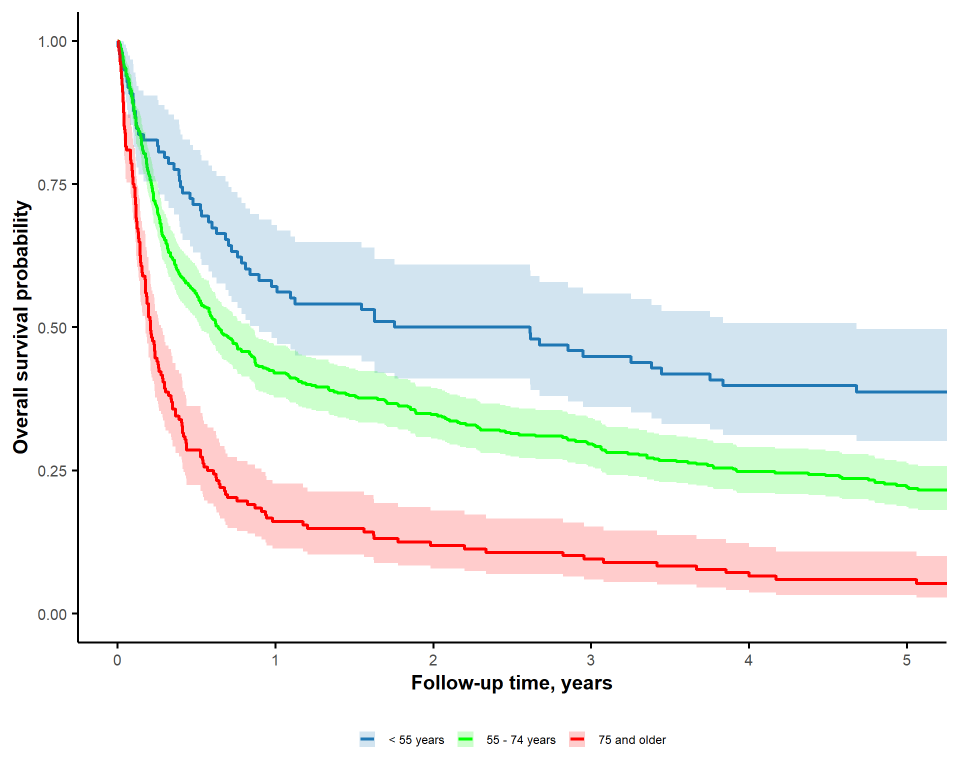

Supplement: Supplementary file 3 — Supporting Information [file JHA2-6-e70021-s003.png]
