# Supplementary material for: Primary Diffuse Large B‐Cell Lymphoma of the Central Nervous System—Outcomes in Finland: A Nationwide Population‐Based Study
Source: EJHaem. 2025 May 28;6(3):e70021. doi: 10.1002/jha2.70021 (PMC12118592; doi:10.1002/jha2.70021)
Supplement: Supplementary file 4 — Supporting Information [file JHA2-6-e70021-s002.docx]

# Supplementary data

| **Supplementary Figure 1.** Overall survival probability in the whole cohort (Supp. Fig. 1A) and in different genders (Supp. 1B).  **Supplementary Figure 2.** Overall survival probability according to two different diagnostic time periods (Supp. Fig 2A) and further stratified according to five different diagnostic time periods (Supp. Fig. 2B).  **Supplementary Figure 3.** Overall survival probability in different diagnostic age groups of <55 years, 55 to 74 years and 75 years and older.  **Supplementary table 1: HRs for excess mortality** | | | |  |  |  |
| --- | --- | --- | --- | --- | --- | --- |
|  | **Unadjusted** | | | **Adjusted** | | |
| **Year of diagnosis** | **HR** | **95% CI** | ***p*-value** | **HR** | **95% CI** | ***p*-value** |
| 1995-2006 | 1.00 (ref) |  |  | 1.00 (ref) |  |  |
| 2007-2009 | 0.92 | 0.72-1.17 | 0.5 | 0.92 | 0.73-1.17 | 0.51 |
| 2010-2012 | 0.8 | 0.63-1.02 | 0.076 | 0.73 | 0.57-0.93 | 0.010 |
| 2013-2015 | 0.81 | 0.64-1.03 | 0.092 | 0.64 | 0.50-0.82 | <0.001 |
| 2016-2018 | 0.63 | 0.50-0.79 | <0.001 | 0.52 | 0.41-0.65 | <0.001 |
